# Bacterial Pericarditis With Underlying Severe Aortic Stenosis and Myelodysplastic Syndrome

**DOI:** 10.7759/cureus.58290

**Published:** 2024-04-15

**Authors:** Kaoruko Aoki, Takatsugu Kajiyama, Goro Matsumiya, Yoshio Kobayashi

**Affiliations:** 1 Department of Cardiovascular Medicine, Chiba University Graduate School of Medicine, Chiba, JPN; 2 Department of Advanced Cardiorhythm Therapeutics, Chiba University Graduate School of Medicine, Chiba, JPN; 3 Department of Cardiovascular Surgery, Chiba University Graduate School of Medicine, Chiba, JPN

**Keywords:** heart failure, pleural pericarditis, myelodysplastic syndrome, bacterial pericarditis, aortic stenosis

## Abstract

We herein describe a case of a 79-year-old male who presented with severe aortic stenosis with myelodysplastic syndrome. He was hospitalized to undergo presurgical evaluation and puncture of pericardiocentesis. After the placement of pericardial drainage, he developed bacterial pericarditis. His heart failure had worsened due to new onset of atrial fibrillation and pericardial constriction. Methicillin-sensitive *Staphylococcus aureus* was identified as the pathogen from the puncture. A pericardial windowing was performed so that his circulatory status was stabilized. An aortic valve replacement as well as resection of pericardial fibrosis was finally performed, and he was discharged without any sequela.

## Introduction

Bacterial pericarditis is a rare but potentially fatal disease. Even in treated patients, the fatality rate is reportedly up to 40% [1]. The major causes of death are cardiac tamponade, sepsis, and constrictive pericarditis. The presence of immunodeficiencies, alcohol dependency, chronic diseases such as rheumatoid arthritis, and cardiac surgery are generally reported as potential risk factors. Because of high mortality, bacterial pericarditis should be promptly treated by a combination of medical and surgical therapies, including pericardial windowing.

## Case presentation

A 79-year-old Japanese male was referred to our hospital because of symptomatic aortic stenosis. He had been treated for hypertension with daily amlodipine 7.5 mg, valsartan 80 mg, and doxazosin 2 mg. He had also been diagnosed with anemia on clinical grounds with no relevant workup for years; however, he had not undergone a detailed examination because of his stable condition. One month before coming to our institution, he developed pitting edema on his lower limbs and exertional shortness of breath for which he visited a local doctor. The examination results indicated severe aortic stenosis and congestive heart failure.

**Figure 1:**
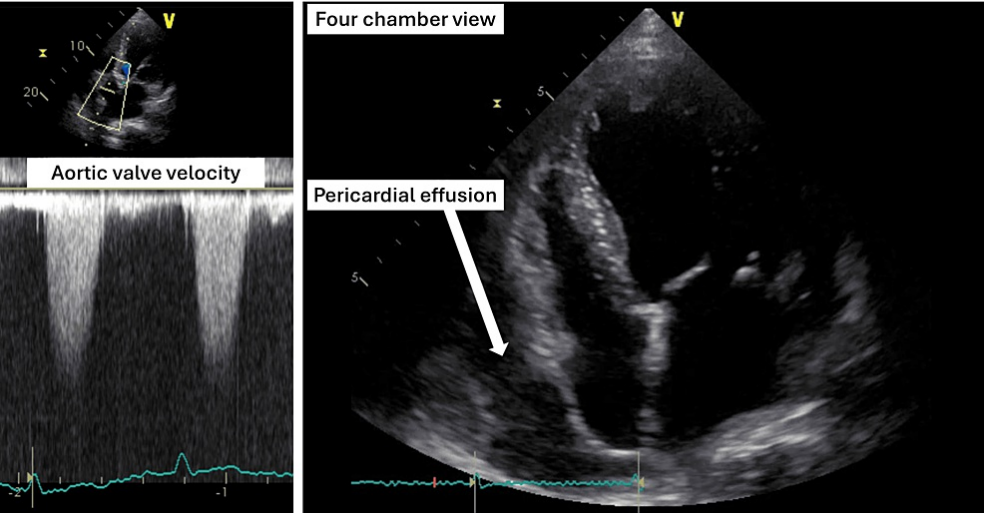
Echocardiogram showing severe aortic valve stenosis (left) and pericardial effusion (right)

**Figure 2:**
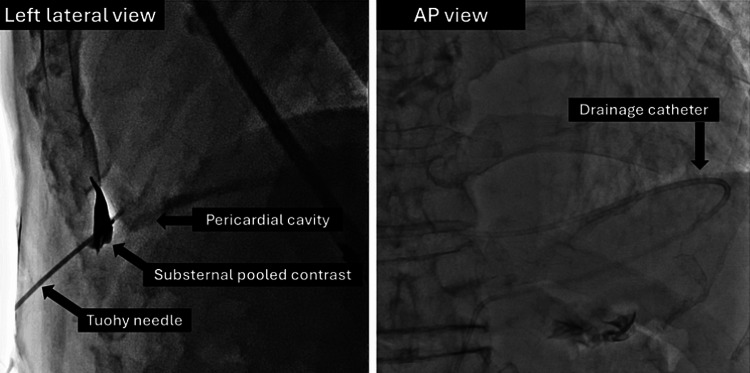
Pericardiocentesis Fluoroscopic image of the substernal puncture (left lateral view on the left and AP view on the right) for performing percutaneous pericardiocentesis

On arrival, he was asymptomatic at rest and his body mass index was 26.0 kg/m^2^. Physical examination revealed conjunctival pallor, a systolic murmur at the secondary aortic area, and bilateral leg edema with stable vital signs (blood pressure = 113/54 mmHg; pulse = regular and 61/min; body temperature = 36.4°C; respiratory rate = 16/min; and oxygen saturation = 95% on room air). Laboratory studies indicated pancytopenia (white blood cell count = 3,500/µL, neutrophils count = 2212/µL, red blood cell count = 2.52×10^6/µL, hemoglobin = 9.3g/dL, and platelet cell count = 107,000/µL) and an increased serum brain natriuretic peptide level (392.1 pg/mL). At this point, there were no signs of local or systemic infection such as a fever, localized redness or swelling, purulent discharge, or elevated white blood cell count and CRP level. Echocardiography revealed severe aortic valve stenosis (peak velocity = 4.8m/s, mean pressure gradient = 50.5 mmHg, aortic valve area = 0.84 cm^2^, and aortic valve area index = 0.47cm^2^/m^2^) and collapse of the right ventricle due to prominent pericardial fluid (Figure 1). The ejection fraction of the left ventricle was preserved. The diastolic function was impaired as follows: (E/A = 121/48 cm/s, deceleration time = 167 ms, E/e' = 22). A following plain CT showed no signs of malignancy. He underwent percutaneous pericardiocentesis to drain the pericardium on Day 2 of his hospitalization. Substernal puncture was performed under echocardiographic and fluoroscopic guidance (Figure 2) with maximum precautions at a catheter laboratory. Serous fluid was obtained. A pathological examination of the effusion was also carried out, which revealed no signs of malignancy. Two days after starting the drainage via a 5-French pigtail catheter, the patient developed a fever of 38.6°C. Laboratory studies indicated an increased CRP level and progression of his anemia (hemoglobin = 8.4g/dL). Considering traumatic pericarditis and hemorrhage associated with the drainage, the catheter was exchanged following another puncture on Day 3. However, the fever persisted and then pus came out of the drain on Day 6. The culture of the first inserted catheter and pus obtained from the puncture site was positive for methicillin-sensitive *Staphylococcus aureus*. A contrast-enhanced CT revealed a pericardial abscess adjacent to the posterior aspect of the left ventricle, with septation. The pericardium was thickened and exhibited contrast enhancement. We diagnosed bacterial pericarditis.

**Figure 3:**
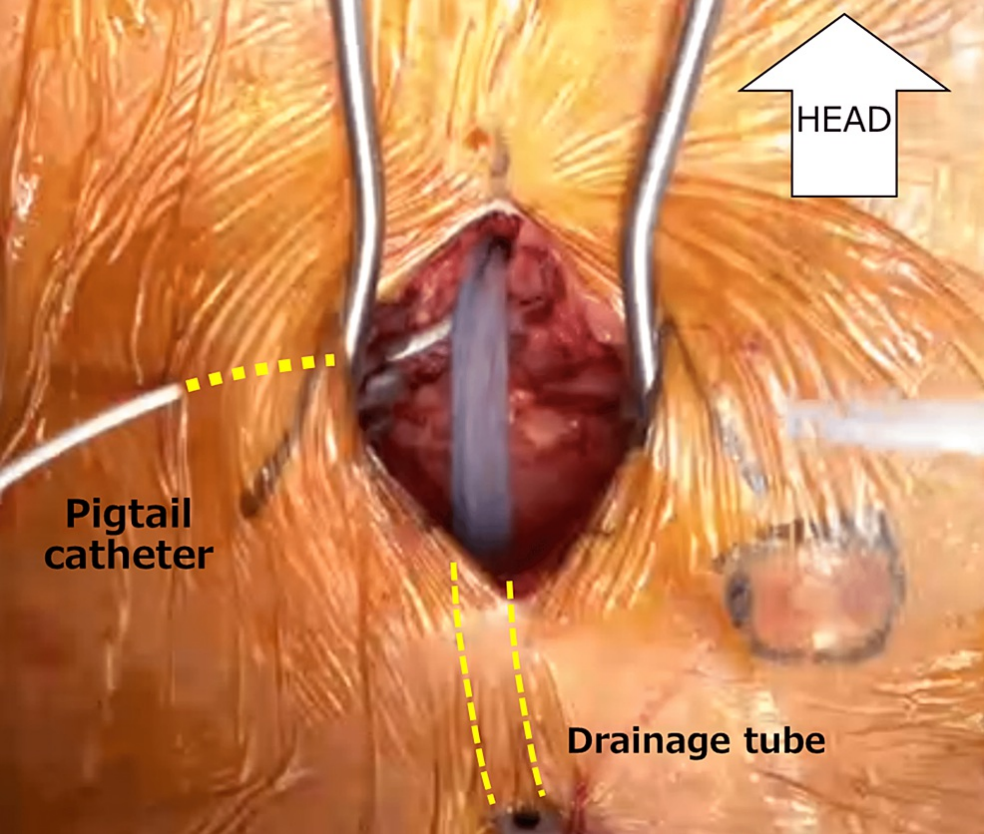
Subxiphoid drainage View during the subxiphoid open-drainage procedure and the location of the drainage tubes. A new pigtail catheter was also inserted in order to wash the pericardium with saline, and the thick tube was exclusively used for drainage.

Despite continuous drainage and intravenous administration of broad-spectrum antibiotics, the patient suffered worsening heart failure as well as elevated lactate levels; therefore, he was admitted to the intensive care unit. We started with ampicillin/sulbactam and then changed to vancomycin. According to the sensitivity test, which revealed the pathogen to be methicilin-sensitive *Staphylococcus aureus*, cefazolin was administered. A balloon aortic valvuloplasty as part of transcatheter aortic valve implantation was judged to be not preferable due to the heavily calcified aortic valve, which may increase the risk of aortic annulus rupture. He also developed atrial fibrillation with a rapid ventricular response along with a poor general status. We performed pericardial windowing to control the infection on Day 11 then two drainage tubes were placed in order to perform repeated flushing with saline (Figure 3).

**Figure 4:**
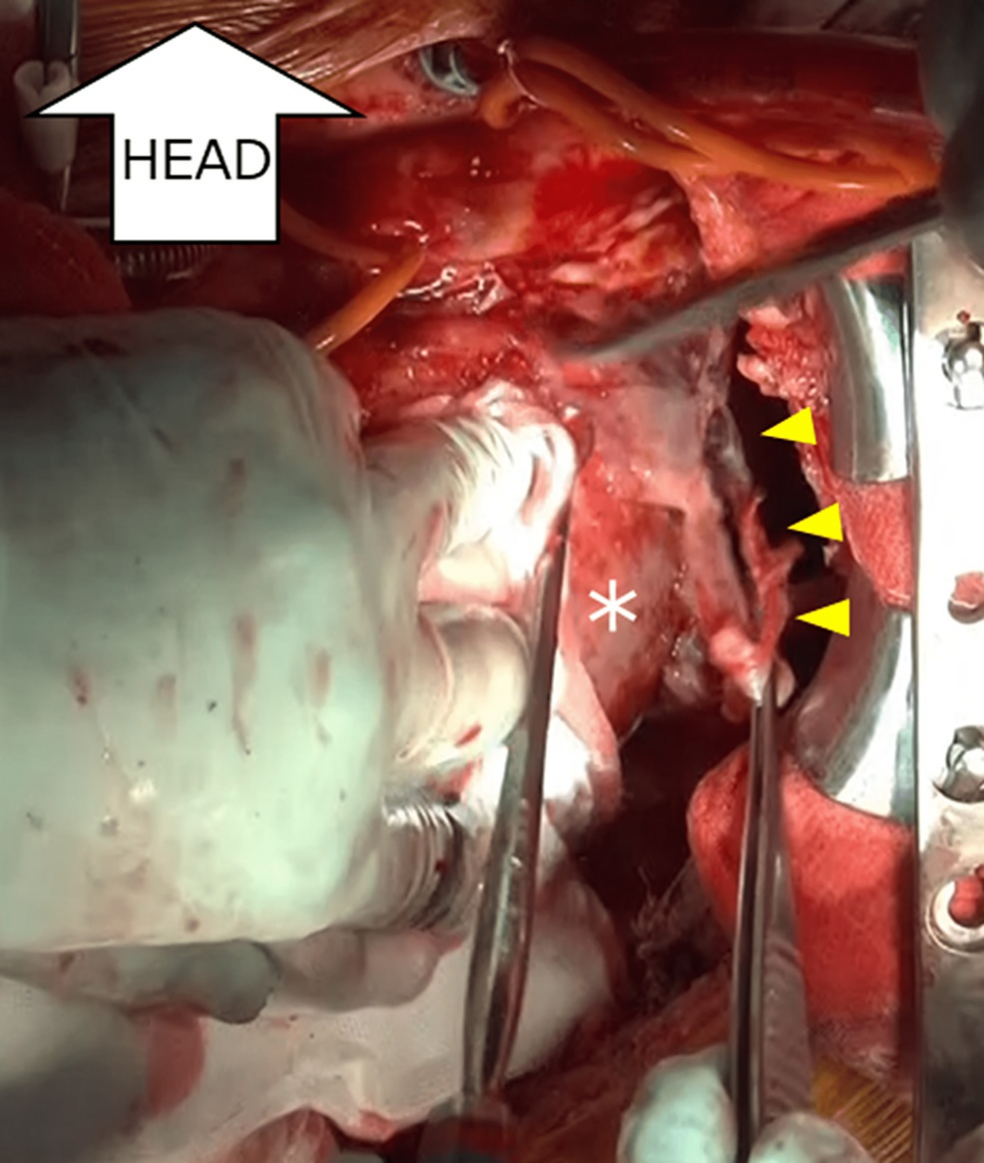
Open-chest drainage Removal of the coagulated effusion from the epicardium. As marked by the yellow indicators, a significant adhesion was observed by traction of the white-colored coagulation from the lateral wall of the left ventricle (white asterisk).

However, his respiratory condition deteriorated and he required ventilator management during this period. We continued the antibiotic therapy and flushing for one week. Methicillin-sensitive *Staphylococcus aureus* was detected from multiple cultures of the pericardial fluid, but all the blood culture tests were negative. Consequently, laboratory studies indicated a decreased CRP level, and the fever came down. However, his heart failure did not improve, presumably because of pericardial constriction. On Day 18, we performed a surgical aortic valve replacement, Cox-maze procedure, and transposition of the omentum. The pericardium was entirely surrounded by a coagulated effusion (Figure 4), which was deemed to be the cause of the diastolic dysfunction of the heart.

The pericardial fluid culture turned negative after the surgery. Postoperative echocardiography did not show any valve seat agitation, obvious adhesion of abnormal structures, or regurgitation of the aortic valve. The patient recovered without any major complications. On the other hand, the progression of pancytopenia led us to perform a bone marrow biopsy, which revealed a diagnosis of myelodysplastic syndrome (MDS). The patient was placed under hematological observation without any additional treatment. After completing the antibiotic treatment, he was discharged on Day 63.

## Discussion

Viral pericarditis, often caused by coxsackieviruses and echoviruses, is the most common type of pericarditis, accounting for about 30% of all pericarditis cases, whereas bacterial pericarditis accounts for only 5% of those cases [1]. Therefore, bacterial pericarditis in adults is rare but always fatal if left untreated. Even in patients who receive standard therapy, the mortality rate reaches up to 40%, mainly due to cardiac tamponade, sepsis, and constrictive pericarditis [2,3]. Constrictive pericarditis in the chronic phase has been reported to occur in about 3% of all bacterial pericarditis cases. However, in this case, bacterial pericarditis acutely caused hemodynamic alteration similar to constrictive pericarditis. Bacterial pericarditis may occur in patients harboring various conditions like immunodeficiencies, chronic diseases such as alcoholism and rheumatoid arthritis, cardiac surgery, and chest trauma. Two large reviews of purulent pericarditis noted that *Staphylococcus aureus* was the most common pathogen, accounting for 20-30% of cases [4,5]. *Streptococcus pneumonia* is also common, and it might be due to the high prevalence of pneumonia as a comorbidity. Infection from gram-positive bacteria accounts for 40-45% of all infections, and infections from multidrug-resistant bacteria are rare [4,6-9]. Effective pericardial drainage and intravenous antibiotics are recommended for purulent pericarditis. Although we started a broad-spectrum antimicrobial therapy, the CRP did not improve. Although the disease can occur in noninvasive conditions and in healthy individuals, drug therapy is not always curative, so surgical treatment is likely to be necessary. Open window surgery is recommended rather than catheter drainage [2]. A subxiphoid pericardiotomy usually allows for better drainage than pericardiocentesis [8]. This procedure can be performed using local anesthesia.

In this case, after pericardial windowing, the inflammatory response gradually improved with repeated daily saline washings. During the valve replacement surgery, adherent fibrin was observed on the posterolateral wall of the heart. Open-chest surgery was deemed to be the most effective to remove extensive fibrin and drain abscesses in this situation. Subsequently, we confirmed the absence of bacterial infection from the pericardial fluid by culture tests and concluded that an effective treatment had been achieved. On the other hand, there are no recommendations for the optimal duration for administering antibiotics to treat bacterial pericarditis cases in the pertinent literature, and it should be determined according to individual clinical course.

In this case, the patient had mild pancytopenia detected in the blood tests from the time of admission. He was diagnosed with MDS by a bone marrow biopsy. MDS is a neoplastic disease characterized by abnormal proliferation and apoptosis of hematopoietic cells and is thought to be caused by abnormalities arising in immature hematopoietic cells. As a result of this neutrophil dysfunction, MDS could cause a compromised condition in our patient who ended up with methicillin-sensitive *Staphylococcus aureus* pericarditis regardless of full precautions in this case [10]. We would like to emphasize that the initial pericardial drainage was performed with full precautions by the physicians and the entire draping of the patient after using both povidone-iodine and chlorhexidine-alcohol for disinfection in the catheter laboratory, in which the air was circulated through Millipore filters. Especially for noninfectious pericardial effusions in immunocompromised patients with hematologic diseases or collagen diseases as in the present case, attention should be paid to the maximum cleanliness during the procedure, and the duration of the drain placement should be kept as short as possible.

## Conclusions

Bacterial pericarditis can occur even after pericardiocentesis, which often requires surgical intervention. Use of maximal sterile barrier precautions is recommended to prevent complications during the catheter drainage of pericardial effusion.
